# Novel venom-based peptides (P13 and its derivative—M6) to maintain self-renewal of human embryonic stem cells by activating FGF and TGFβ signaling pathways

**DOI:** 10.1186/s13287-020-01766-9

**Published:** 2020-06-18

**Authors:** Rui Ma, Zhili Ren, Bin Li, Shirley W. I. Siu, Guokai Chen, Hang Fai Kwok

**Affiliations:** 1 Institute of Translational Medicine, Faculty of Health Sciences, University of Macau, Avenida de Universidade, Taipa, Macau SAR; 2 Cancer Centre, Faculty of Health Sciences, University of Macau, Avenida de Universidade, Taipa, Macau SAR; 3 Centre of Reproduction, Development & Aging, Faculty of Health Sciences, University of Macau, Avenida de Universidade, Taipa, Macau SAR; 4 Department of Computer and Information Science, Faculty of Science and Technology University of Macau, Avenida de Universidade, Taipa, Macau SAR

**Keywords:** Venom peptide, Self-renewal, Embryonic stem cell, Peptide modification, Pluripotency

## Abstract

**Background:**

In our previous study, a venom-based peptide named Gonearrestide (also named P13) was identified and demonstrated with an effective inhibition in the proliferation of colon cancer cells. In this study, we explored if P13 and its potent mutant M6 could promote the proliferation of human embryonic stem cells and even maintain their self-renewal.

**Methods:**

The structure-function relationship analysis on P13 and its potent mutant M6 were explored from the molecular mechanism of corresponding receptor activation by a series of inhibitor assay plus molecular and dynamics simulation studies.

**Results:**

An interesting phenomenon is that P13 (and its potent mutant M6), an 18AA short peptide, can activate both FGF and TGFβ signaling pathways. We demonstrated that the underlying molecular mechanisms of P13 and M6 could cooperate with proteoglycans to complete the “dimerization” of FGFR and TGFβ receptors.

**Conclusions:**

Taken together, this study is the first research finding on a venom-based peptide that works on the FGF and TGF-β signaling pathways to maintain the self-renewal of hESCs.

## Introduction

The advantages of venom-based peptides from animals such as scorpions, spiders, and snakes are their stable structures and high specificities for target molecules due to a hundred million years of adaptive evolution [1, 2]. Recently, our research group developed a high-throughput screening platform and identified a venom peptide named Gonearrestide (18AA, MW2194, also called P13) from an in-house scorpion venom library [3]. In our previous study, we demonstrated that P13 could effectively inhibit the proliferation of colon cancer cells via triggering the cell cycle arrest in the G1 phase. It is well known that the relationship between the structure and function of ion channels, such as Voltage-sensitive Na^+^ channels and K^+^ channels, can be studied by using venom-based peptides [4–6]. However, the relationship between venom-based peptides and receptors involved in stem cell self-renewal or differentiation has rarely been reported.

Human embryonic stem cell (hESC) therapy has been proposed for regenerative medicine and tissue replacement after injuries or diseases such as Parkinson’s disease, spinal cord injury, and diabetes [7–9]. Although stem cells have extensive self-renewal potential, they require high regenerative conditions to maintain pluripotency in vitro [10]. The mTeSR™1 and Essential 8™ (E8) media are the main products on the market for the growth and expansion of human pluripotent stem cells (hPSCs), ES cells, and induced pluripotent stem cells (iPS cells) [11, 12]. Chen et al. developed the E8 medium that has few components and easy quality control as compared to mTeSR™1 (19 components) [12, 13]. The E8 medium is Xeno- and feeder-free and has eight components: DMEM/F12, FGF2, TGFβ1, insulin, l-ascorbic acid, selenium, NaHCO3, and transferrin. The Essential 6™ Medium (E6) is a medium based on the E8 medium but without two components, FGF2 and TGFβ1, which supports the reprogramming of somatic cells and the differentiation of hPSCs.

Interestingly, when P13 was added to the E8 medium to culture human ES cells, P13 did not inhibit the growth of human ES cells. Instead, it promoted their massive expansion. The FGF and TGFβ signaling pathways have been reported to promote the self-renewal mechanism of hESCs (Supplementary Fig. S1) [14–16]. The TGFβ signal-activated SMADs directly bind with the NANOG proximal promoter and upregulate the expression of NANOG [14]. Pluripotency-associated key genes such as NANOG, OCT4, and SOX2 maintain the long-term self-renewal ability of the hESCs [17]. Through this study, we revealed that P13 upregulated the protein expression of p-Erk1/2 and p-Smad2 and activated the expression of OCT4, SOX2, and NANOG, the important factors for maintaining the self-renewal ability of the hESCs.

However, maintaining the self-renewal mechanism of the hESCs requires a relatively higher concentration of P13, which is not ideal for the hESC culture application. To find a potent isoform of P13, the P13 “binding domain” was modified, and the in-depth study of the relationship between structure and function was performed from starting at the molecular mechanism of corresponding receptor activation. One of the P13 mutants, M6, showed a much higher proliferative activity. Furthermore, two FGFR inhibitors and three TGF-β receptor inhibitors were used to confirm the signaling pathway mechanism involved in P13 and its mutant M6 (Supplementary Fig. S1). In addition, molecular modeling and simulations were used to predict the binding modes of P13 and M6 to FGFR and provide insights on the molecular mechanism to FGFR activation induced by the peptides in the hESCs. After a series of inhibitor assay and molecular dynamics simulation studies, we found that underlying molecular mechanisms of P13 and its potent mutant M6 can cooperate with proteoglycans to complete the “dimerization” of FGFR and TGFβ receptors. As a result, we found that P13 is the first venom-based peptide that acts on both the TGF-β and FGF signaling pathways to promote the self-renewal of the hESCs.

## Materials and methods

### Human ES cell culture

The human ES cells (hESCs, H1 from WiCell Research Institute, Inc., Madison, WI, http://www.wicell.org) were maintained in an E8 medium using matrigel-coated tissue culture plates [12]. The E8 medium had eight components: DMEM/F12, l-ascorbic acid-2-phosphate magnesium (64 mg/l), sodium selenium (14 μg/l), insulin (19.4 mg/l), NaHCO3 (543 mg/l), transferrin (10.7 mg/l), and the FGF2 (100 μg/l) and TGFβ1 (2 μg/l) growth factors. The cells were passaged every 3–4 days once the cells reached 80–85% confluency by using the EDTA method in the presence of Y-27632 as previously described [18]. The E8 medium had to be changed every day. The hESCs with normal karyotypes were used in less than the passage of P50.

### Survival assay

The human ES cells were dissociated with TrypLE (Gibco) for 5–8 min at room temperature and neutralized with the medium containing 0.5% BSA. The cell counts were determined using a BD Accuri C6 flow cytometer and normalized to the original cell number that was plated.

### The modification of P13

As per the Cardin-Weintraub model [-X-B-B-X-B-X-], in which B is a basic residue and X is a hydropathic residue [19], P13 was mutated to synthesize its derivate P13-M5 (Supplementary Table S1). The heparin-binding affinity of the observed basic amino acid arginine was 2.5 times more than lysine [20–22]. So, the two lysines of P13-M5 were mutated into two arginines to get the mutant P13-M6. To investigate the function of the N-terminal P13 key amino acids, two other mutants, P13-M10 and P13-M11, were synthesized based on P13-M6 (Supplementary Table S1).

### The immunostaining of human ES cells treated with fluorescently labeled P13/M6

The human ES cells were cultured on sterilized coverslips, and a 12-well plate was coated with matrix gel at a concentration of 1 mg/ml for 1–2 h. The cells were washed with the DMEM-F12 medium after 24 h, and the E8 medium was changed and treated with fluorescently labeled peptide for 24 h. Then, the cell membrane was stained using the CellMask™ Deep Red plasma membrane (Thermo Fisher) and incubated at 37 °C for 10 min. The staining solution was removed, and 200 μl 4.0% formaldehyde was added to the medium and incubated at 37 °C for 10 min. The medium was then removed, and the cells were washed with 1X PBS three times. The cells were incubated with 10 μl DAPI (DNA stain) and the mounting medium for 1 min, and the cover glass was mounted. The coverslip was sealed with nail polish to prevent drying. The samples were imaged using a Carl Zeiss Confocal LSM710 microscope.

### The expression of p-Erk1/2 and p-Smad2 in the human ES cells treated with P13, M6, and small molecular inhibitors

The human ES cells (H1) were seeded into the 12-well plate with an E8 medium. The medium was changed to E6 after 24 h. The human ES cells were incubated at 5% CO2 and 37 °C for 20–24 h, the P13 and M6 peptides were added, and the cells were harvested using RIPA buffer after 1 h.

The human ES cells (H1) were seeded into the 12-well plate with an E8 medium. The medium was changed to E6 after 24 h. The human ES cells were incubated at 5% CO2 and 37 °C for 20–24 h, and certain concentrations of FGFR inhibitors—AZD4547 [23], PD173074 [24], and TGFβ receptor inhibitors LDN193189 [25], SB431542 [26], and A8301 [27, 28]—were added according to the layout (Under the conditions of TGFβ receptor inhibitors, three 12-well plates were arranged in the experiment (a total of 22 conditions in Fig. 5b). The three 12-well plates were LDN193189, SB431542, and A8301 inhibitor, respectively. Each plate also had E6 without inhibitor as a blank control; under the conditions of FGFR inhibitors, the experiment arranged two 12-well plates (a total of 14 conditions in Fig. 5c). One plate was AZD4547 inhibitor and the other was PD173074, and each plate had E6 without inhibitor as a blank control; under the conditions of AZD4547 and A8301 double inhibitors, a 12-well plate was arranged in the experiment (a total of 12 conditions in Fig. 5d). A 12-well plate was arranged for the verification experiments of single inhibitor AZD4547 and A8301 and validation of simultaneous addition of dual inhibitors AZD4547/A8301, and E6 without inhibitor as a blank control.) and incubated for 3 h; then certain concentrations of P13 and M6 peptide were added, and the cells were collected using RIPA buffer after 1 h.

### Western blotting

For the western blotting of soluble proteins, the human ES cells were harvested using RIPA buffer with benzonase, protease inhibitors, and phosphatase inhibitors. The protein concentration was measured using the Pierce™ BCA Protein Assay Kit (Thermo Fisher). Twelve percent SDS-PAGE gel was used, and the proteins in the gel were transferred onto a nitrocellulose membrane. The membrane was blocked using 5% defatted milk in a 1xPBST buffer for an hour at room temperature with shaking, incubated with the primary antibody at 4 °C overnight, and then with the secondary antibody-HRP for 1 h. The PVDF membrane was washed three times with 1xPBST for 10 min at the end of each incubated antibody step. The Pierce™ ECL Western Blotting Substrate was used to detect chemiluminescence (Thermo Fisher) on the ChemiDoc Imaging System (Bio-Rad).

### Total RNA extraction and qPCR to check self-renewal markers

The hESCs’ RNA was extracted using the RNeasy Mini Kit (QIAGEN). One thousand nanograms per tube of isolated RNA was reverse transcribed into cDNA using the High-Capacity cDNA Reverse Transcription Kit (Thermo Fisher). The qPCR was performed on the BioRad CFX96 Touch Real-Time PCR using the PowerUp SYBR Green Master Mix (Thermo Fisher). The observed primer sequences are listed in Supplementary Table S2. All the experiments were performed in triplicates, and the relative gene expression was normalized with GAPDH.

### CDOCKER of A8301 inhibitor to TGFβ receptor

CDOCKER is a CHARMM-based molecular dynamics (MD) scheme to dock ligands into a receptor binding site by adopting high-temperature kinetics to search for flexible conformational spaces of ligand molecules and simulating the annealing to map each conformation at the active receptor site. The TGF-beta receptor type-1 (PDB ID: 3TZM) was selected as the acceptor, the small molecule inhibitors contained in the crystal structure were deleted, and the site sphere was selected from the PDB site records. Then, A8301 was chosen as the ligand. The parameters set were as follows: 1000 random conformation dynamics steps, 1000 k dynamics target temperature; 2000 simulated annealing heating steps, 700 k heating target temperature, 5000 cooling steps, and 300 k cooling target temperature; forcefield: CHARMM [29]. The Discovery Studio v17.2.0 commercial software was used.

### Molecular dynamics (MD) simulations

Molecular dynamics (MD) simulations were used to investigate the effects of the two venom-based peptides on the structure and dynamics of the FGFR dimer. We used the PDB entry 1FQ9 (3 Å resolution) [30] as starting structures for the receptor, its ligand, and the co-factor. This PDB contains two FGFs, two FGFRs, and two heparan sulfate (HS) molecules bound in a stable ternary complex. On the other hand, the initial coordinates of P13 and M6 peptides were obtained by prediction using the PEP-FOLD3 server [31]. The C-termini of both peptides were amidated in accordance with the experimental forms. The simulation systems with four different setups were constructed: the *crystal* 2:2:2 FGF:FGFR:HS complex, the *apo* 2:2 FGFR:HS complex, and the 2:2 FGFR:HS complex bound with chains of P13 or M6. We believe that multiple peptides were required to exert effects on the biological function of the protein, so we performed preliminary studies for systems with 10, 20, and 30 chains of P13 and M6 with short MD simulations. These peptides were randomly placed in the solvent phase of the system and allowed to equilibrate around the protein complex. Initially, position-restraints were applied on all heavy atoms of the protein complex to prevent the change in the receptor conformation. As the number of peptides contacted with the complex was converged after 200 ns, the position restraints were removed and the systems were simulated for another 200 ns. Based on the observed better stability of the protein receptor (Supplementary Fig. S2), we focused our study on the 20-chain peptide systems and extended these simulations until 500 ns.

Topology files for the simulation systems were generated using the CHARMM-GUI web interface [32] with the following options: (1) fix the missing inner residues in the FGFR chain B (residue 293 to 307); (2) model four suggested disulfide bonds (178 and 230, 277 and 341 of the FGFR chain A and B); (3) glycosylation of both heparin molecules; (4) add counter ions to neutralize the system; and (5) solvate the entire complex with water molecules in a rectangular box. The prepared systems contain approximately 200,000 atoms in a box of 13.0 × 13.0 × 13.0 nm^3^ (Supplementary Table S3). All simulations were performed under periodic boundary conditions using GROMACS version 5.0.7 [33]. The proteins, peptides, and heparins were modeled by the CHARMM36m force field [34] and the water molecules by TIP3P [35]. Short-range interactions were cutoff at 1.2 nm with the use of the switching potential for van der Waals interactions starting at 1.0 nm. Long-range interactions were treated by particle mesh Ewald [36] with a Fourier spacing of 0.12 nm. Bonds with a hydrogen atom were constrained using the LINCS [37] and SETTLE [38] algorithms, so a time step of 2 fs could be used. Production simulations were performed in the isothermal-isobaric (NPT) ensemble. The Nosé-Hoover thermostat [39] was used at 300 K with a coupling constant of 1.0 ps. The pressure was maintained at 1 atm with the Parrinello-Rahman barostat [40] and coupling constant of 5.0 ps. All initial systems were firstly equilibrated by the canonical ensemble (NVT) with velocity generation. Production trajectories were generated by NPT ensemble, and coordinates were saved every 10 ps for analysis.

## Results

### The proliferation of human ES cells treated with venom-based peptide P13

**Fig. 1:**
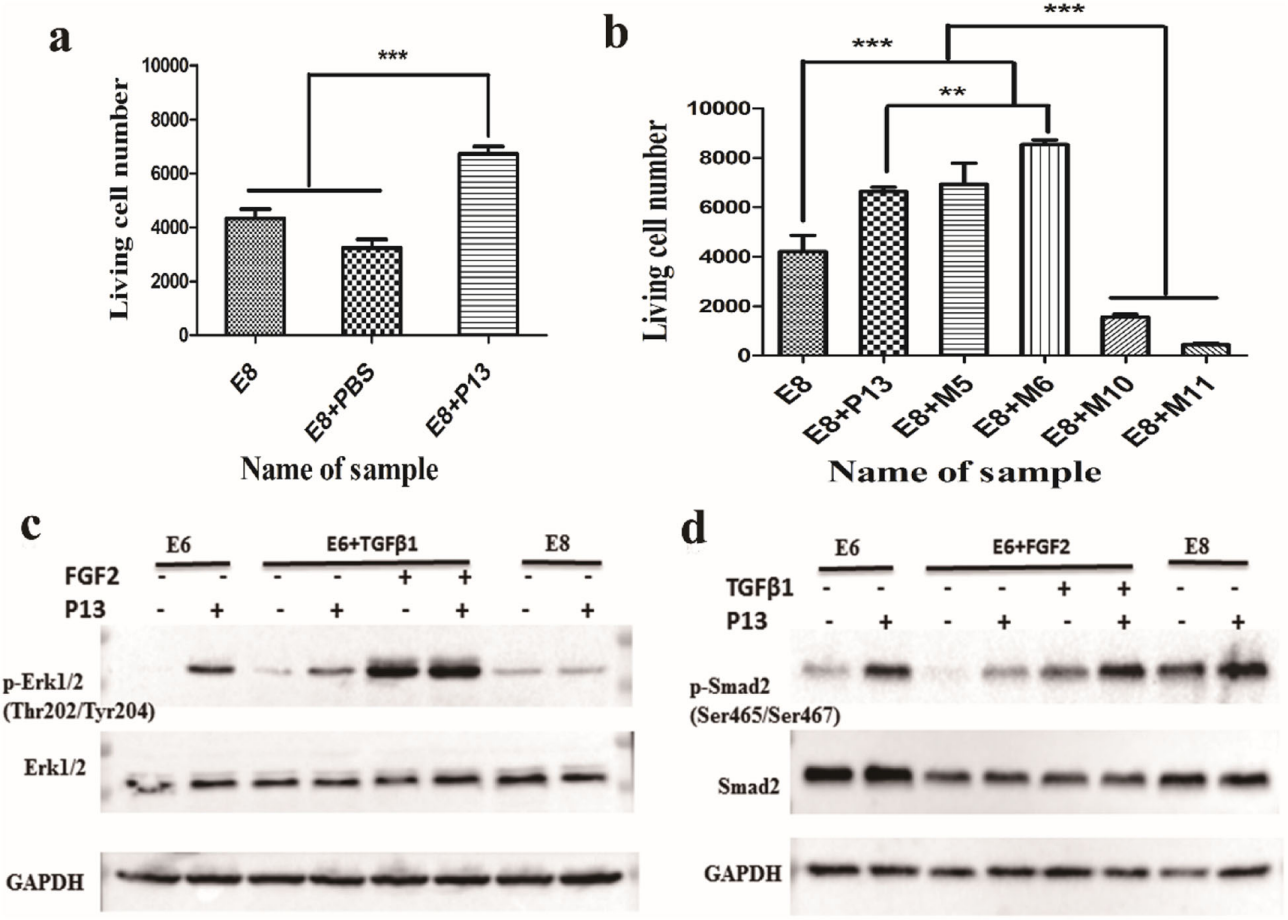
The survival ability and expression levels of p-Erk1/2 and p-Smad2 in human ES cells. **a** The survival ability of the human ES cells treated with venom peptide P13; the concentration of P13 was 200 μM/L; 1 × PBS was the solvent of the same volume used to dissolve the peptides. The statistical significances are marked by asterisks—*p < 0.05, **p < 0.01, and ***p < 0.001—between the two indicated groups. **b** The survival ability of the human ES cells treated with P13 and its mutants (M5, M6, M10, and M11); the concentration of P13 and its mutants was 200 μM/L. **c** The expression of the total/phosphorylation Erk1/2 in different culture conditions (E6, E6 + TGFβ1, and E8) when treated with P13 (left). **d** The expression of total/phosphorylation Smad2 in different culture conditions (E6, E6 + FGF2, and E8) when treated with P13 (right); the concentration of FGF2 was 100 μg/L, and the concentration of TGFβ1 was 2 μg/L.

When venom-based peptide P13 was added to the E8 medium-cultured human ES cells, we found that it did not inhibit the growth of human ES cells but promoted the proliferation of human ES cells. After the human ES cells were grown in a 48-well culture dish for 24 h, the human ES cells were dissociated. The cell counts were determined using a BD Accuri C6 flow cytometer. Using one-way analysis of variance (ANOVA), we found that the number of human ES cells that survived in the E8 medium with P13 was significantly higher than that of the human ES cells cultured only in the E8 medium (Fig. 1a).

### The activation of p-Erk1/2 and p-Smad2 in human ES cells treated with P13

The human ES cells were cultured under three different culture conditions (E6, E6 + FGF2, and E8) and these conditions treated with P13 to observe the differences in the expressions of p-Erk1/2 (Fig. 1c, left); and under three different culture conditions (E6, E6 + TGFβ1, and E8) and these conditions treated with P13 to observe the differences in the expressions of p-Smad2 (Fig. 1d, right). Figure 1c (left) shows that the observed expression level of p-Erk1/2 was significantly upregulated in the E6 medium stimulated by P13 as compared to the E6 control medium. Compared to the human ES cells in the E6 + TGFβ1 medium, the expression level of p-Erk1/2 was upregulated in the E6 medium with P13 and/or FGF2. Compared to the human ES cells in the E8 medium, the expression level of p-Erk1/2 was not significantly upregulated in the E8 medium with P13. Figure 1d (right) shows that the observed expression level of p-Smad2 was significantly upregulated in the E6 medium stimulated by P13 as compared to the E6 control medium. Compared to the human ES cells in the E6 + FGF2 medium, the expression level of p-Smad2 was upregulated in the E6 medium with P13 and/or TGFβ1. Compared to the human ES cells in the E8 medium, the expression level of p-Smad2 was upregulated in the E8 medium with P13.

### The verification of human ES cells’ self-renewal ability when treated with P13

**Fig. 2:**
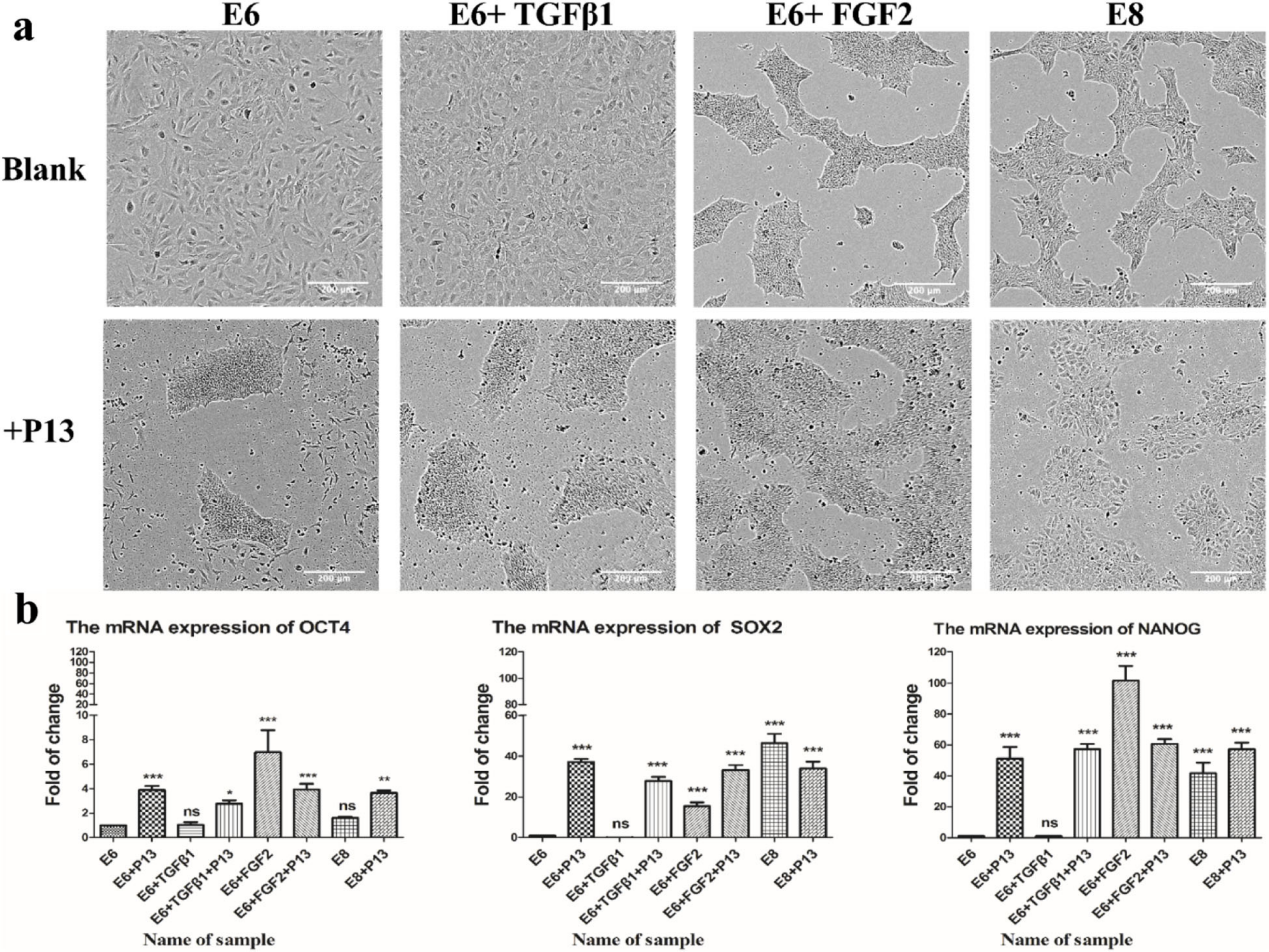
The human ES cells’ morphology and self-renewal capacity when treated with P13. **a** These images of the human ES cells were taken after passaging five times (magnification, 10×); the concentration of P13 was 200 μM/L, and the blank represents the 1 × PBS solvent for dissolving the peptides. **b** The qPCR results of POU5 F1, SOX2, and NANOG of the human ES cells in different culture conditions and treated with P13

According to the preliminary results, four different media (E6, E6 + TGFβ1, E6 + FGF2, and E8) were prepared, and peptide P13 was added to them; in total, eight different media were prepared. The human ES cells were serially passaged five times under eight different conditions to detect the differences in morphology and pluripotency. Figure 2a shows the observed morphological differences after five passages. The colony-forming characteristics of the human ES cells disappeared in the E6 and E6 + TGFβ1 media. However, the human ES cells still maintained their colony morphology under the E6 + P13 and E6 + TGFβ + P13 conditions after five passages. In the E6 + P13 medium, the human ES cells formed a mixed state of colonies and dispersed cells. Then, when P13 was separately added to the E6 + FGF2 and E8 media, the human ES cells showed no significant differences in the morphology after continuous culture for five generations.

The quantitative polymerase chain reaction (qPCR) method was used to examine the self-renewal markers of human ES cells. The mRNA of OCT4, SOX2, and NANOG was examined for differential expressions under eight different conditions (Fig. 2b). Low mRNA expression levels of OCT4, SOX2, and NANOG were maintained in E6 and E6 + TGFβ1 as compared to the other conditions, which was consistent with the results we observed in the morphological Fig. 2a. Compared to the E6 and E6 + TGFβ1 media, the OCT4, SOX2, and NANOG of the human ES cells were upregulated in the other six conditions. A one-way ANOVA (***p < 0.001) showed that the mRNA expression of NANOG was significantly upregulated compared to the E6 medium.

### The survival assay of P13 mutants

The survival assay used to discover P13 was also used to screen for its mutants. When the amino acids at positions 12 and 13 were exchanged, the mutant P13-M5 (K12E/E13K) was obtained (Supplementary Table S1). The number of survival human ES cells in the E8 + M5 medium showed a slight increase, but there was no significant difference to the number of cells in the E8 + P13 medium. When the lysine at positions 13 and 14 in M5 was replaced by arginine, the mutant P13-M6 (K12E/E13R/K14R) was obtained. A one-way ANOVA showed that the number of survival human ES cells in the E8 + M6 medium was significantly higher than those in the E8 + P13 medium (Fig. 1b). Concurrently, using the M6 as a template, two key amino acids (cysteine and aspartic acid at the N-terminus of P13) were also mutated to obtain two mutants, P13-M10 and P13-M11. Interestingly, the number of human ES cells that survived in the E8 + M10/M11 media was significantly lower than those in the E8 medium (Fig. 1b). The primary structure and molecular weight of P13 and its mutants are listed in Table S1.

### The verification of the human ES cells’ self-renewal ability when treated with M6

**Fig. 3:**
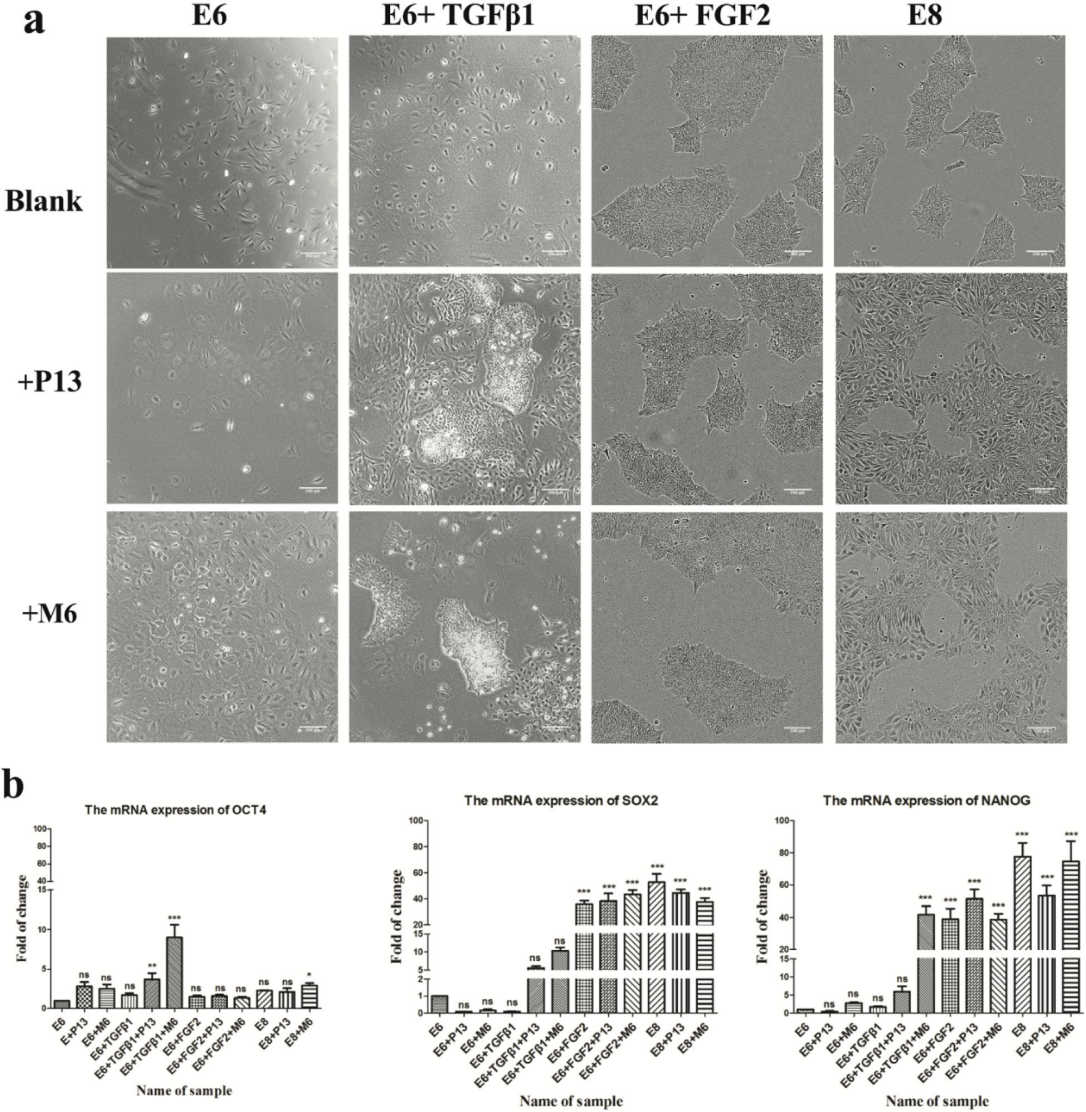
The human ES cells’ morphology and self-renewal ability when treated with M6. **a** The microscope images were taken after the human ES Cells were passaged five times, and P13 was used as a control (magnification, 10×); the concentration of P13 and M6 was 100 μM/L, and the blank represents the 1 × PBS solvent used to dissolve peptides. **b** The qPCR results of OCT4, SOX2, and NANOG of the human ES cells in different culture conditions treated with P13/M6

Based on the preliminary results, four different media (E6, E6 + TGFβ1, E6 + FGF2, and E8) were prepared, and peptide M6 (P13 as a control) was added to them as an additional component; then, twelve different media were prepared in total. The human ES cells were serially passaged five times under 12 different conditions to detect the differences in morphology and pluripotency. Figure 3a shows the morphological difference after five passages. The colony-forming characteristic of the human ES cells totally disappeared in the E6, E6 + P13, E6 + M6, and E6 + TGFβ media. However, the human ES cells maintained their colony-forming morphology in the E6 + TGFβ + P13 and E6 + TGFβ + M6 conditions after five passages compared to E6 + TGFβ medium. In the E6 + TGFβ + P13 and E6 + TGFβ + M6 medium conditions, the human ES cells showed a mixed state of colonies and dispersed cells. P13 and M6 were then separately added to the E6 + FGF2 and E8 media, and the human ES cells showed no significant differences in their morphologies after five generations of continuous culture.

The qPCR assay was used to examine the self-renewal markers of human ES cells. The mRNA of OCT4, SOX2, and NANOG was examined for differential expression under 12 different conditions (Fig. 3b). Low mRNA expression levels of OCT4, SOX2, and NANOG were maintained in E6, E6 + P13, E6 + M6, and E6 + TGFβ1 as compared to the other conditions, which were consistent with the results observed in the above morphological Fig. 3a. Compared to the E6 + TGFβ1 medium, the OCT4, SOX2, and NANOG of the human ES cells were upregulated in the E6 + TGFβ1 + p13 and E6 + TGFβ1 + M6 conditions. A one-way ANOVA showed that the expression levels of SOX2 and NANOG were significantly upregulated in the E6 + FGF2, E6 + FGF2 + P13, E6 + FGF2 + M6, E8, E8 + P13, and E8 + M6 media as compared to the E6 medium (***p < 0.001).

### Localization of peptides in human ES cells to verify its cell-penetration ability

**Fig. 4:**
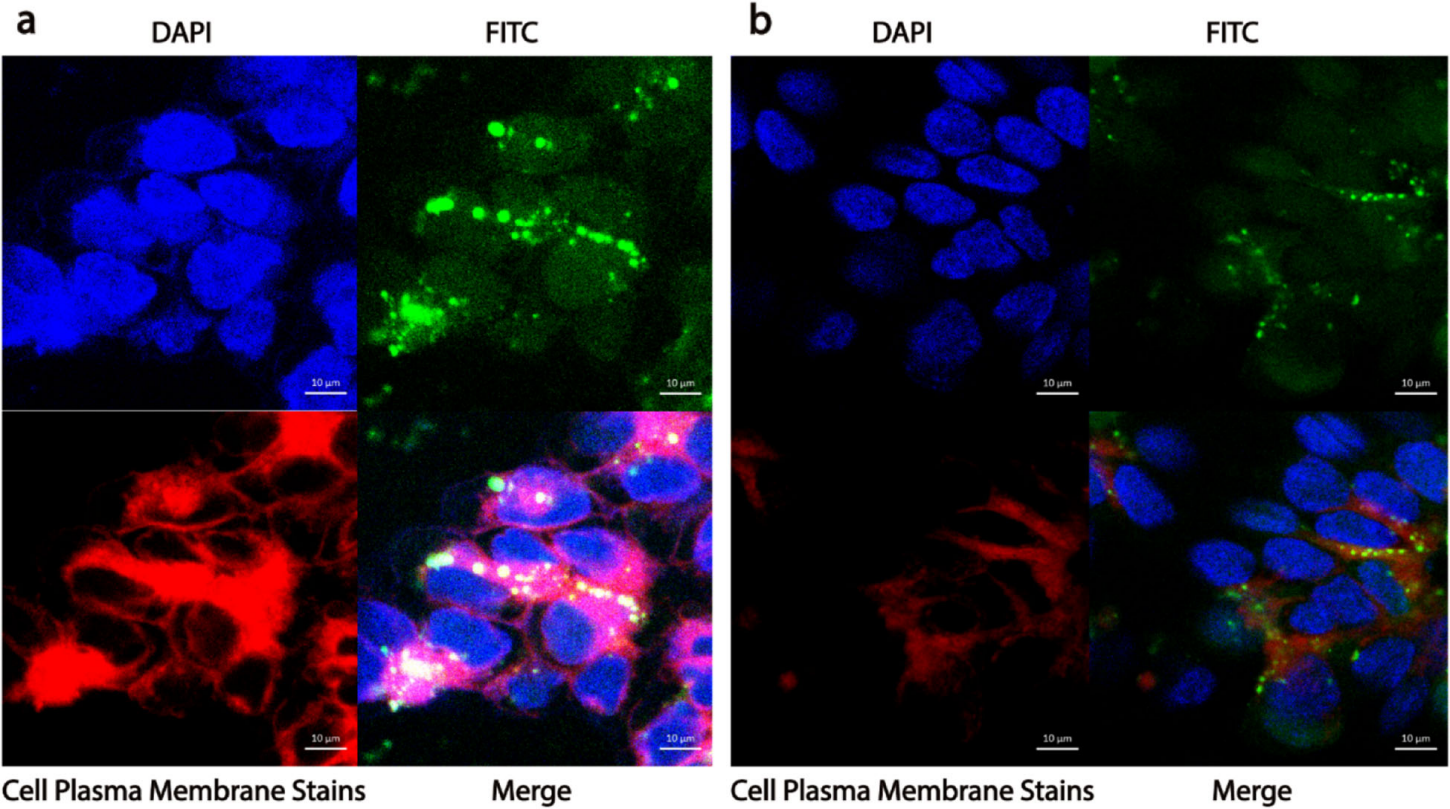
The location of P13 and its mutant M6, labeled by FITC, in the membrane of human ES cells. Confocal microscope figures with 100× oil magnification (**a** P13-FITC and **b** M6-FITC)

Some short peptides can act as cell-penetrating peptides (CPPs) that penetrate cell membranes to perform their functions or serve as carriers to transport certain molecules into cells [41]. The purpose of this experiment was to determine whether P13 and M6 can enter cells to perform their respective functions. The human ES cells were treated with P13 and M6, each labeled by FITC fluorescence, for 24 h, and the location of peptides was confirmed through confocal microscopy by the immunostaining of nuclear dye combined with cytoplasmic membrane dye. As can be seen in Fig. 4, the blue fluorescence represented the nucleus of the embryonic stem cells, red fluorescence represented the cell membrane, green fluorescence-labeled P13 and M6 were bound to the plasma membrane, and no green fluorescence-labeled P13 and M6 are found in the cytoplasm. This indicated that P13 and M6 do not belong to the CPP family.

### The validation of p-Erk1/2 and p-Smad2 expression in human ES cells treated with M6 without small molecular inhibitors

**Fig. 5:**
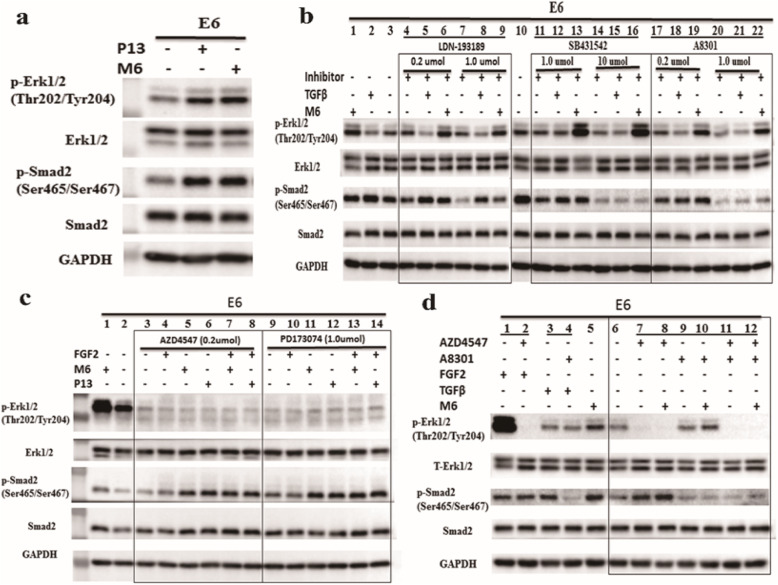
The expressions of p-Erk1/2 and p-Smad2 in the human ES cells treated with M6 without/with small molecular inhibitors. **a** The expression of total/phosphorylation Erk1/2 and total/phosphorylation Smad2 when treated with P13 and its mutant M6 without any inhibitor. **b** The expressions of the TGFβ signaling pathways after being treated with TGFβ receptor inhibitors (LDN-193189, SB431542, and A8301); the concentration of SB431542 was 1.0 and 10 μmol, and the concentration of A8301/LDN-193189 was 0.2 and 1.0 μmol. **c** The expression of the FGFR pathways after being treated with FGFR receptor inhibitors (AZD4547 and PD173074); the concentration of AZD4547 was 0.2 μmol, and the concentration of PD173074 was 1.0 μmol. **d** The expressions of the FGF and TGFβ signaling pathways after being treated with the FGFR and TGFβ receptor dual inhibitors (0.2 μmol AZD4547 and 1.0 μmol A8301)

Based on the structural modification of P13 at the active site, we obtained a mutant, M6, with better proliferation activity. First, we compared the differences in the expression of p-Erk1/2 and p-Smad2 in the human ES cells stimulated by P13 and M6. Compared to the human ES cells in the E6 medium, the expression levels of p-Erk1/2 and p-Smad2 were significantly upregulated in the E6 medium with P13 and M6, respectively (Fig. 5a). However, compared to the human ES cells in the E6 medium with P13, the expression levels of p-Erk1/2 and p-Smad2 were not significantly upregulated in the E6 medium with M6.

### The molecular modeling of the receptor kinase domain bound to a small molecular inhibitor

**Fig. 6:**
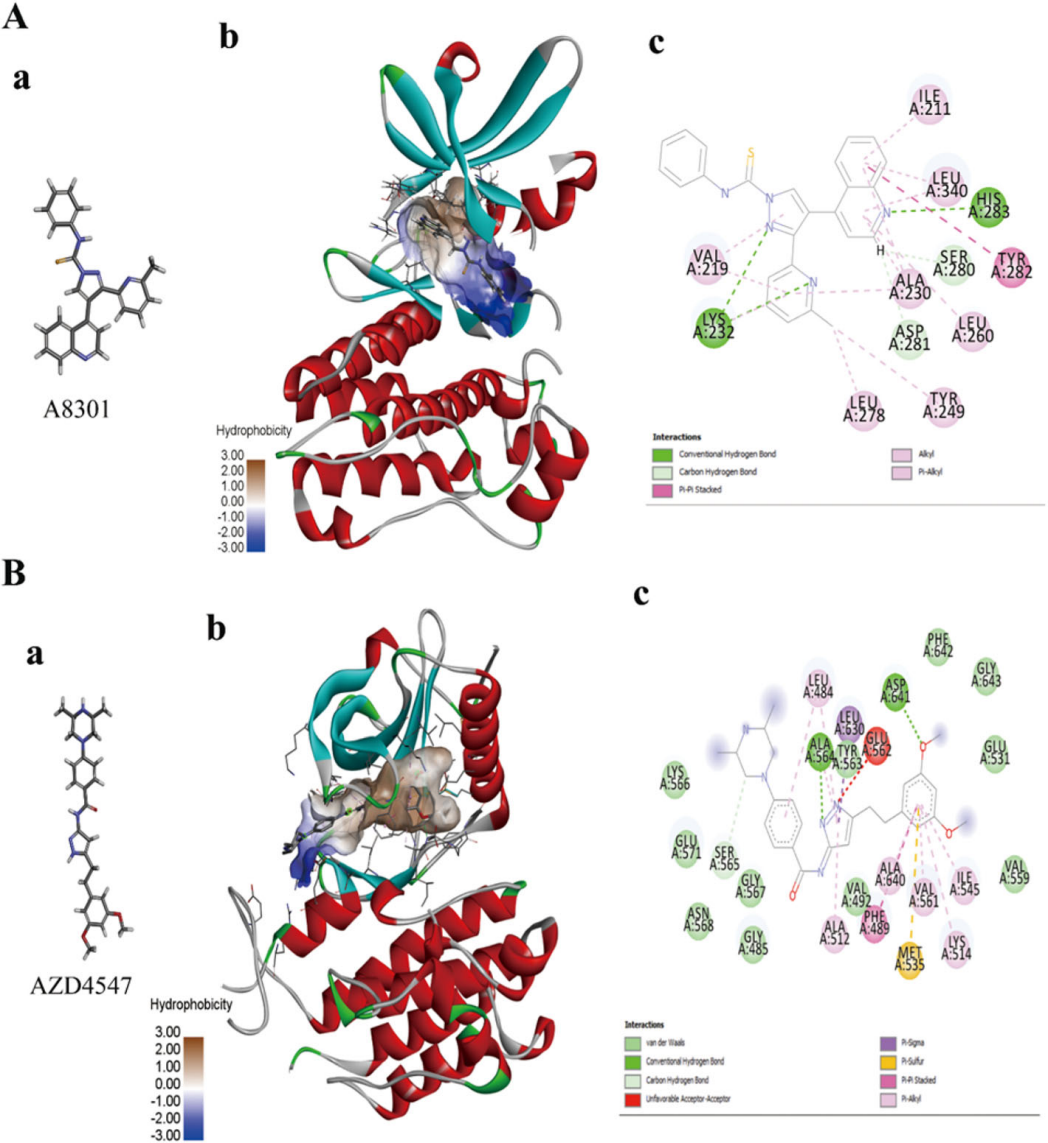
The molecular modeling of the kinase domain treated with small molecular inhibitors. **A** The solid ribbon modeling of the FGFR kinase domain bound to AZD4547 based on the PDB ID 4WUN: (a) represents the 2D structure of AZD4547; (b) represents the AZD4547 bound to the pocket of the FGFR intracellular kinase domain; and (c) is a 2D model of the interaction between AZD4547 and FGFR in the active molecular window. **B** The solid ribbon modeling of the TGFβ receptor kinase domain bound to A8301 based on the PDB ID 3TZM: (a) represents the 2D structure of A8301; (b) shows the A8301 bound to the pocket of the TGFβ receptor intracellular kinase domain; and (c) is a 2D model of the interaction between A8301 and TGFβ in the active molecular window

The PDB ID 3TZM represents the intracellular kinase domain of TGFβ receptor type 1 [27]. The 3TZM is actually a three-dimensional crystal structure that includes the small molecular inhibitor SB431542 and the TGFβ receptor. CDOCKER deleted the SB431542 and docked the A8301 into the FGFR intracellular kinase domain. The small molecular inhibitor A8301 bound the intracellular kinase domain binding pocket of TGFβ receptor type 1 (Fig. 6A). Figure 6A (c) shows a 2D model of the interaction between A8301 and TGFβ receptor type 1 in the active molecular window. The amino acids interacting with the receptor and ligand were labeled. The small molecule inhibitors bound to the kinase domain of the receptor, effectively inhibiting the phosphorylation of downstream molecules. The PDB ID 4WUN represents the intracellular kinase domain of FGFR1 [42]. A small molecular inhibitor, AZD4547, was bound to the binding pocket of the FGFR intracellular kinase domain (Fig. 6B). Figure 6B (c) shows a 2D model of the interaction between AZD4547 and FGFR in the active molecular window, and the interacting amino acids between the receptor and ligand have been labeled.

### The expression of p-Erk1/2 and p-Smad2 in human ES cells treated with M6 with small molecular inhibitors

The human ES cells treated with TGFβ family receptor inhibitors LDN189193, SB431542, and A8301 to study the expression levels of p-Erk1/2 and p-Smad2. LDN193189 has the ability to inhibit ALK1 (ACVRL1), ALK2 (ACVR1A), ALK3 (BMPR1A), and ALK6 (BMPR1B) (IC50 = 0.8, 0.8, 5.3, and 16.7 nM, respectively) and is a potent inhibitor of the bone morphogenetic (BMP) pathway [25]; SB431542 inhibits ALK4 (ACVR1B) and ALK5 (TGFβR1) (IC50 = 140 nM, 94 nM, respectively) and is a selective and potent inhibitor of the TGF-β/Activin/NODAL pathway [26]. A8301 is a selective inhibitor of the type I activin/nodal receptor ALK4, TGF-β type I receptor ALK5 kinase, and type I nodal receptor ALK7 (ACVR1C) (IC50 = 45, 12, and 7.5 nM, respectively) [27, 28]. As shown in the lanes 3–9 of Fig. 5b, 1.0 μmol LDN189193 could inhibit the expression of p-Smad2 as compared to the control E6 medium (lane 3) and 0.2 μmol LDN189193 could not inhibit the expression of p-Smad2; however, compared to the stem cells in the E6 medium with 1.0 μmol LDN189193, the expression of p-Smad2 was upregulated in the respective E6 media treated with TGFβ and M6. Lanes 11–16 (SB431542) and 17–22 (A8301) show that 1.0 μmol SB431542 and 0.2 μmol A8301 could not inhibit the expression of p-Smad2. However, a higher concentration of SB431542 (10 μmol) and A8301 (1.0 μmol) could significantly inhibit the expression of p-Smad2; the expression of p-Erk1/2 was upregulated in the E6 medium with M6, but the upregulated expression of p-Erk1/2 could not be observed in the E6 medium with TGFβ.

The human ES cells were treated with FGF family receptor inhibitors AZD4547 and PD173074 to observe the expression levels of p-Erk1/2 and p-Smad2. AZD4547 [23] is a strong FGFR1/2/3 inhibitor (IC50 = 0.2, 2.5, and 1.8 nM, respectively) with weaker activity against FGFR4. Docetaxel (AZD4547) has entered a phase II/III trial to treat patients with stage IV squamous cell lung cancer (https://clinicaltrials.gov). PD173074 is a potent FGFR1 inhibitor (IC50 = 25 nM) in cell-free assays [24]. Lanes 2–8 in Fig. 5c show that 0.2 μmol AZD4547 could inhibit the expression of p-Erk1/2 compared to the control E6 medium (lane 2) and the E6 media treated with P13, M6, and/or FGF2. However, the expression of p-Smad2 was upregulated in the E6 media treated with P13, P13 + FGF2, M6, and M6 + FGF2 as compared to the control E6 medium and E6 medium with 0.2 μmol AZD4547. Lanes 9–14 in Fig. 5c show that 1.0 μmol PD173074 could inhibit the expression of p-Erk1/2 as compared to the control E6 medium and the E6 media with P13, M6, and/or FGF2. However, the expression of p-Smad2 was upregulated in the E6 media with P13, P13 + FGF2, M6, and M6 + FGF2 as compared to the control E6 medium and E6 medium with 1.0 μmol PD173074.

The human ES cells were treated with the FGF receptor inhibitor AZD4547 (0.2 μmol) and/or TGFβ receptor inhibitor A8301 (1.0 μmol) to study the expression of p-Erk1/2 and p-Smad2, as shown in Fig. 5d. The lanes 1–2 validate the function of the FGF2 and FGF receptor inhibitor AZD4547; FGF2 upregulated the expression of p-Erk1/2, and AZD4547 inhibited the expression of p-Erk1/2, even in the E6 medium with FGF2. The lanes 3–4 validate the function of the TGFβ1and TGFβ receptor inhibitor A8301; TGFβ1 upregulated the expression of p-Smad2, and A8301 inhibited the expression of p-Smad2, even in the E6 medium with TGFβ1. The lanes 7–10 validated the expression difference between p-Erk1/2 and p-Smad2 in the single and double inhibitors (FGF and TGFβ) of the human ES cells treated with M6. The lanes 7–8 show that the expression of p-Erk1/2 was inhibited by 0.2 μmol AZD4547, even in the E6 medium with M6, and the lanes 9–10 show that the expression of p-Smad2 was inhibited by 1.0 μmol A8301, even in the E6 medium with M6. However, the lanes 11–12 show that the expressions of p-Erk1/2 and p-Smad2 were inhibited by the dual-inhibitors 0.2 μmol AZD4547 and 1.0 μmol A8301, even in the E6 medium with M6.

### Molecular dynamics (MD) simulations

**Fig. 7:**
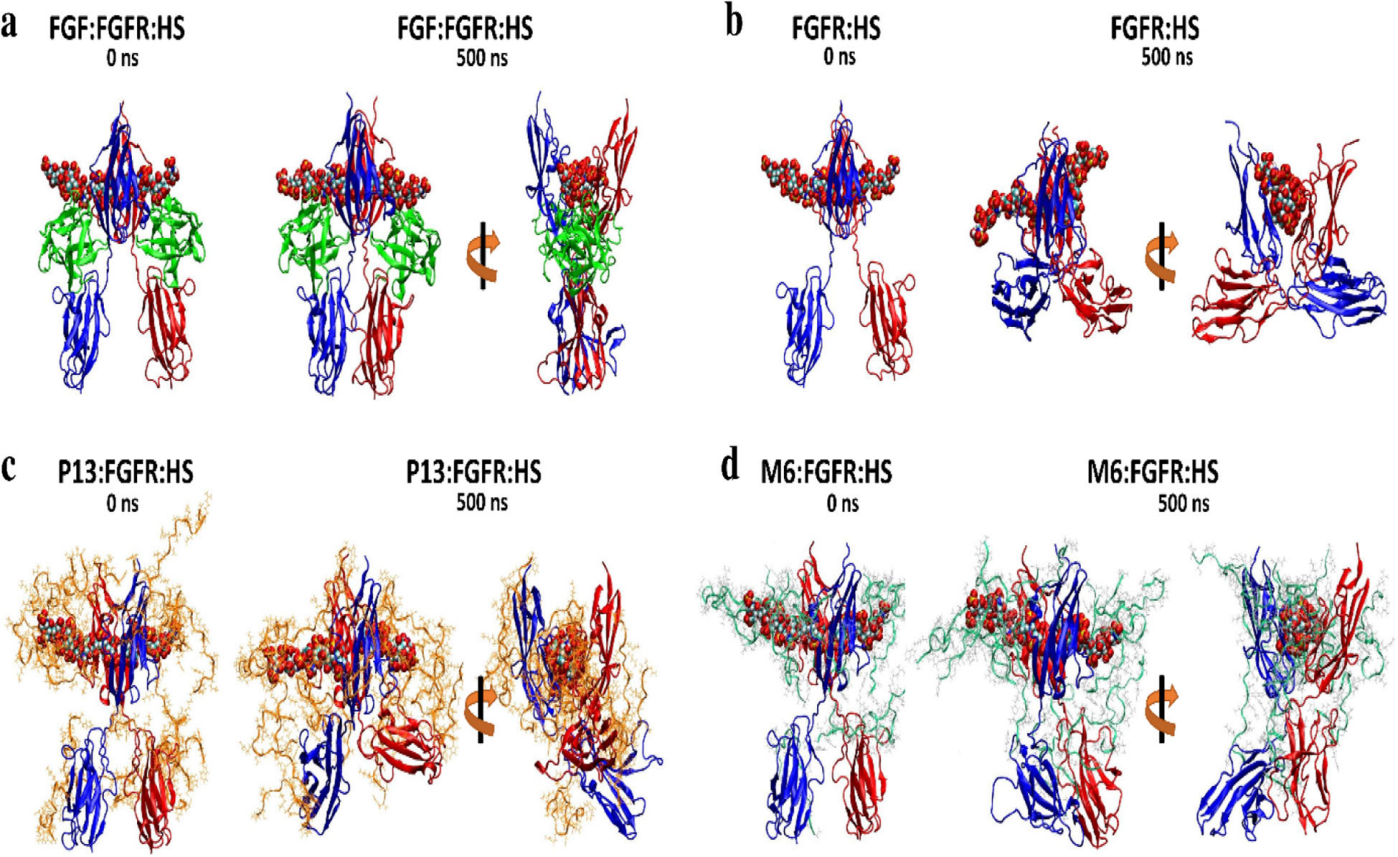
Simulation snapshots of four types of the simulation system. **a** The crystal FGF:FGFR:HS complex. **b** The apo FGFR:HS complex. **c** The FGFR:HS complex with 20 chains of P13. **d** The FGFR:HS complex with 20 chains of M6. Few peptides that are not in contact with protein were removed for clarity

Our biological experimental results suggest that both P13 and M6 are promising alternatives to FGF for activating the FGFR protein and inducing the intracellular downstream signaling with the latter exhibited better proliferation activity. To understand the biological function of the two peptides at the molecular level, we modeled peptide binding with the 2:2 FGFR:HS complex by classical MD simulations. Twenty chains of peptides were added to the solvent phase of the system and allowed to equilibrate around the protein complex. Structures and dynamics of the protein complex in the presence of peptides were analyzed and compared to the *native* 2:2:2 FGF:FGFR:HS (FGF-bound) and *apo* 2:2 FGFR:HS (FGF-free) complexes. Figure 7 shows the initial and final snapshots of the 500-ns simulations of four types of systems. As clearly seen, multiple peptides are associated with the complex in both the upper D2 and lower D3 domains of the receptor. M6 appears to bind more strongly and tightly with the complex while P13 exhibits looser binding with frequent attachment and detachment events in the course of a simulation. To determine factors to the peptide binding, we computed the non-bonded interaction energies between the peptide and complex from the Coulombic and Lennard-Jones potentials. As shown in Supplementary Fig. S3, the binding of the peptide to the complex is dominantly driven by electrostatic interactions. A comparison of the energy values of M6 to those of P13, we see that there are pronounced electrostatics and van der Waals interactions of HS-peptide (dE = − 774.6 and − 109.2 kJ/mol, respectively). On the other hand, P13 shows stronger binding to the protein in electrostatic interaction (dE = − 441.9 kJ/mol). The total non-bonded interaction energy of M6 is − 8142.2 kJ/mol compared to P13 of − 7690.3 kJ/mol, which gives a difference of − 451.9 kJ/mol.

**Fig. 8:**
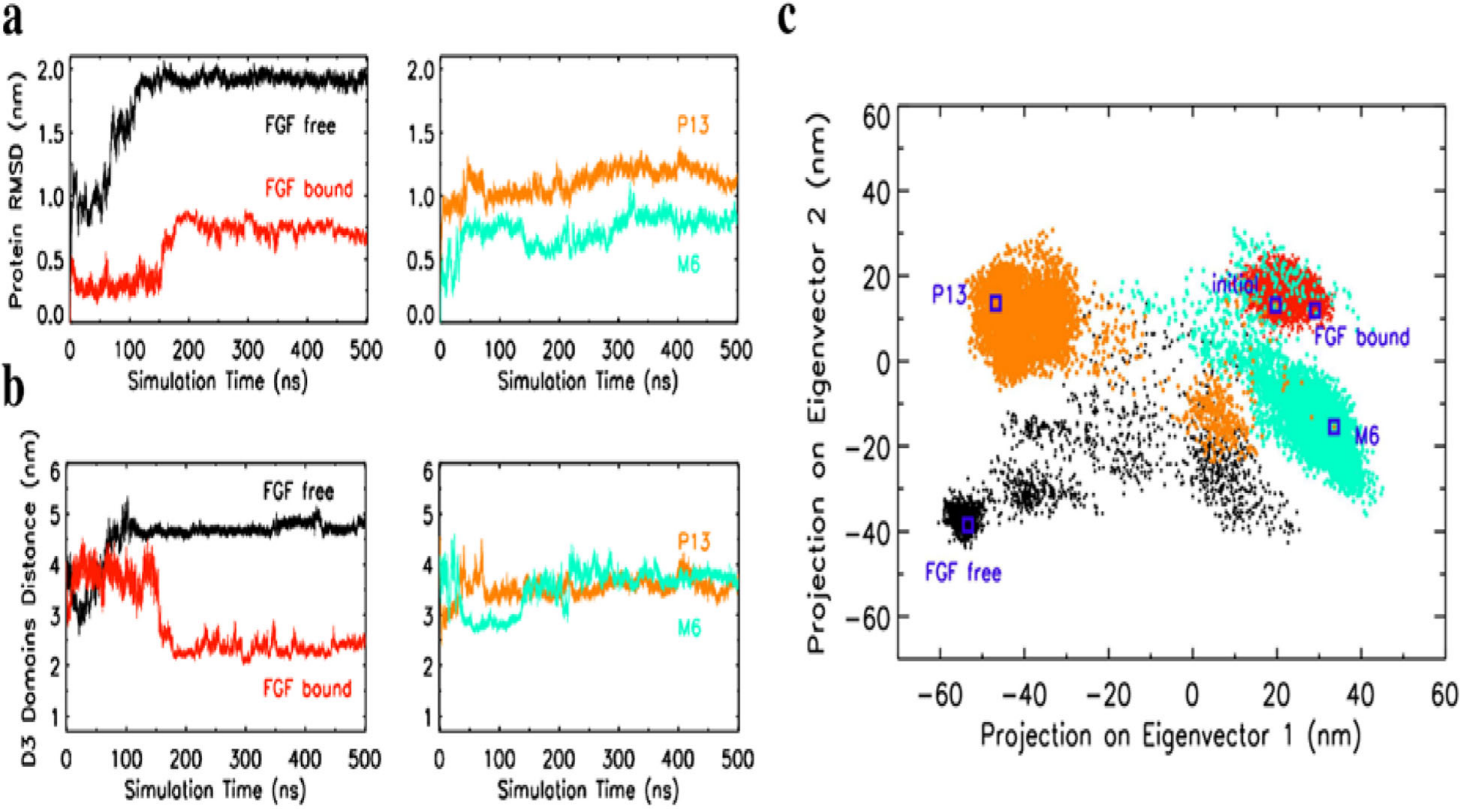
Analysis of structural changes of FGFR under different ligand conditions: FGF-free, FGF-bound, P13, and M6. **a** The root-mean-squared deviation (RMSD) of the protein backbone in reference to its initial structure. **b** The center-of-mass separation distance between the D3 domains of the two FGFR chains. **c** Two-dimensional projection of the four combined trajectories (FGF-free, FGF-bound, P13, and M6) on the first and second PCA modes computed from the coordinates of FGFR backbone atoms. The square symbols indicate positions of protein conformations in terms of PCA coordinates for the initial (all trajectories have the same initial protein conformation) and the final state (500 ns) of each trajectory. Color codes for trajectory: FGF-bound (red), FGF-free (black), P13 (orange), M6 (cyan)

Consequently, the differential bindings of M6 and P13 to the complex induced different changes in the structure of the complex. With M6, the structure of the FGFR:HS complex resembles the equilibrated FGF-bound structure where the D2 and D3 domains are stretched and the D3 domains of the two chains stay in the proximal distance. In contrast, with P13, the structure of the FGFR:HS complex resembles closer to the equilibrated FGF-free structure that it has undergone a large conformational change to adopt a strikingly different fold upon the loss of its native ligand. A comparison of the root-mean-squared deviation (RMSD) and D3 separation distance in Fig. 8a and b reveal that the change in the complex structures of both P13 and M6 occurred almost immediately but the changed structures reached stability at 300 ns. The close interactions of the D3 domains in both P13 and M6 are stable, though in a slightly larger separation distance (center of mass of the two domains is approx. 3 nm versus 2 nm in the equilibrated FGF-bound state of the crystal simulation). This discovery is consistent with the result that D3 of the activated two FGF receptors gets closer by using the FRET-based technique [43].

We further analyzed the conformational change of the complex in the course of simulation by principal component analysis (PCA). We concatenated the four simulation trajectories to be compared, namely FGF-bound, FGF-free, P13, and M6, and computed the covariance matrix of positional fluctuations of protein backbone atoms. The matrix was diagonalized; the eigenvectors and associated eigenvalues representing the axes of atomic motions and mean square fluctuations along these axes were obtained. Figure 8c shows the 2-dimensional projection of the simulation trajectories onto the first and second PCA modes, axes of the largest and second-largest atomic motions of the protein. The result of PCA analysis shows that the conformational change of the protein from the initial state to the active state (equilibrated FGF-bound form) involves only short-distance atom movement whereas the change to the inactive state (equilibrated FGF-free form) involves large-scale motion corresponding to the closing action of the D2-D3 domains of the two chains. The projection data reveals that upon binding of M6 peptides, the protein maintained similar conformational states as the initial and FGF-bound forms for some time, then it switched to a conformational state that is close to the FGF-bound form. Interestingly, with P13, the protein underwent relatively larger motion to settle on conformational states that are intermediary between the initial and FGF-free states.

## Discussion

Self-renewal is the process of cell division with the maintenance of pluripotency and is enabled to eliminate the differentiation-inducing signal of mitogen-activated protein kinase [44]. The Erk1/2 signaling stimulated by FGFs and the SMAD2/3 signaling stimulated by TGFβ are critical for the maintenance of the stem cell self-renewal ability [12, 45]. The crystal structure of FGF2 binding the extracellular region of FGFR was first determined at 2.8 Å resolution in 1999 [46]. The same research group analyzed the crystal structure of a ternary FGF-FGFR-Heparin complex (PDB ID: 1FQ9) in the second year and discussed a dual role of heparin [30]. Subsequent mutation analysis and related experimental evidence further prove the rationality of the 1FQ9 crystal structure [47, 48]. Therefore, this study used 1FQ9 as a template for molecular dynamics analysis. In 2008, the crystal structure of two molecules of TGFβ3 bound to heterotetrameric TGFβ RI/ TGFβ RII (2PJY) was determined at 3.0 Å resolution [49]. However, the crystal structure of TGFβ bound to the corresponding receptor is a complicated heterotetramer, which leads to relatively few studies so far, and more experimental evidence is needed to prove its crystal structure rationality. How do the 18AA short toxin peptide P13 and M6 play a similar role in FGF2 (16.4 kDa) and TGFβ1 (25.6 kDa disulfide-linked homodimer)? We try to discuss this question by exploring the activation mechanisms of their corresponding receptors.

**Fig. 9:**
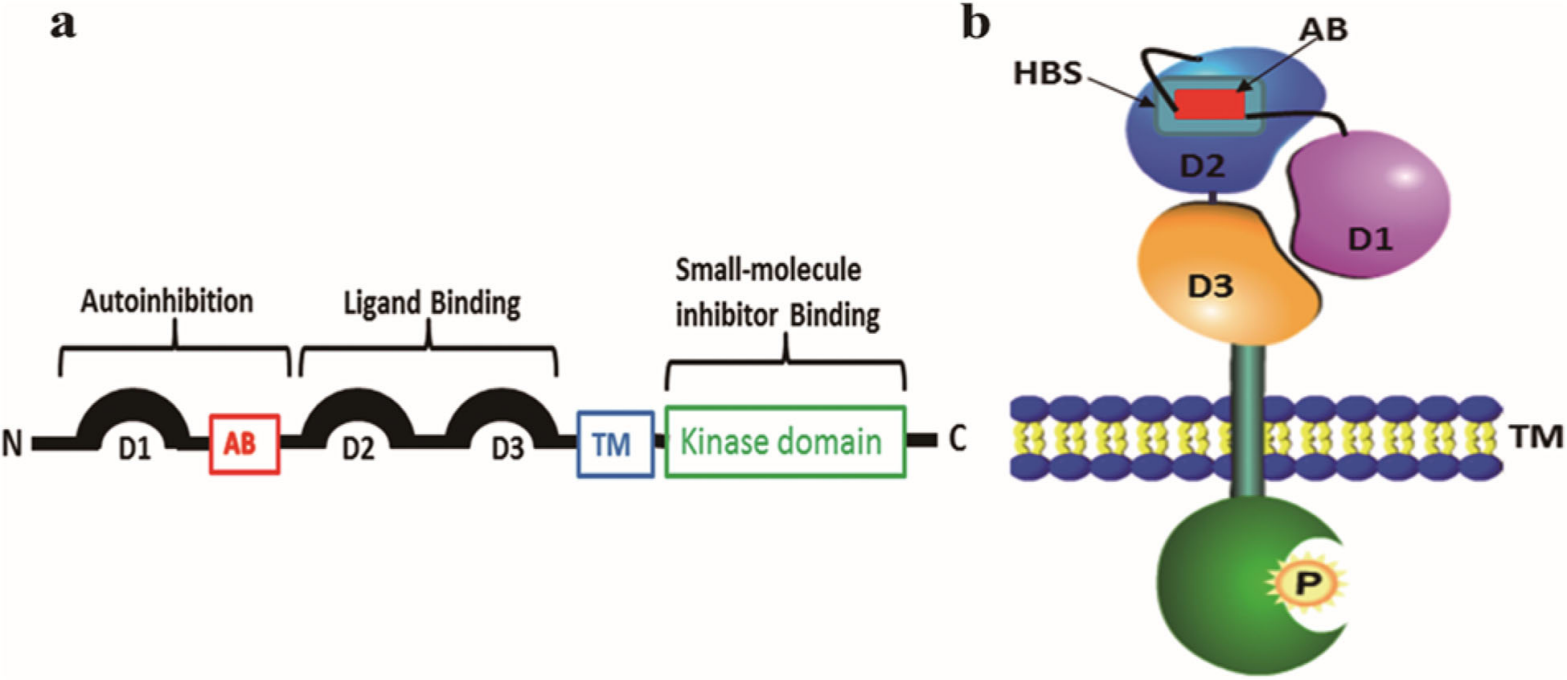
Schematic diagram and autoinhibition model of FGFR. **a** Schematic diagram of FGFR: The “AB” in red stands for acid box, and TM represents the transmembrane domain. Kinase domain phosphorylates downstream molecules and can be inhibited by small-molecule inhibitors. The extracellular region of FGFR is involved in receptor autoinhibition, and the ligand-binding is indicated. The N- and C-terminals are labeled N and C, respectively. **b** Autoinhibition model of FGFR: The HBS is the heparin-binding site. AB binds to the HBS of D2 to inhibit the activation of FGFR. Figure source adopted from Mohammadi et al. [47, 48] and modified by Rui MA

Fibroblast growth factor receptors (FGFRs) are transmembrane proteins composed of three domains: an extracellular ligand-binding region, a single transmembrane portion, and an intracellular kinase domain [50] (Fig. 9a and Supplementary Fig. S4). The extracellular region consists of three immunoglobulin-like regions: D1, D2, and D3. An acid box (AB) exists between the D1 and D2 regions. The D1 and acid box play key roles in the self-regulation of FGFR molecules, and D2 and D3 are ligand-binding regions [47]. Kinase domain phosphorylates downstream molecules and can be inhibited by small-molecule inhibitors such as AZD4547 and PD173074. The D2 region includes a heparin-binding site. FGFRs have their own set of intramolecular regulatory mechanisms—“autoinhibition” (Fig. 9b) [51]. D1 competitively binds to a ligand-binding site formed by D2 and D3. At the same time, the acid box binds to the positively charged “canyon” within the same receptor, which is also the heparin-binding site (18AA in FGFR1: KMEKKLHAVPAAKTVKFK (six lysine residues) and 18AA in FGFR2: KMEKRLHAVPAANTVKFR (three lysine residues and two arginine residues)) (Supplementary Fig. S4) [46]. When D1 and the acid box occupy their intramolecular binding sites, the FGFR monomer adopts a “closed” and auto-inhibited configuration; however, when FGF and heparan sulfate proteoglycans (HSPGs) individually compete for the ligand-binding site and acid box, the FGFR in the closed configuration is opened and activated. From the above-unbiased evidence, we speculated that venom peptides P13 (18AA: WCYKLPDRVSIKEKGRCN (three lysine residues and two arginine residues)) and its mutant M6 (18AA: WCYKLPDRVSIERRGRCN (one lysine residues and four arginine residues)) have the ability to compete for the acid box to relieve the autoinhibition of FGFR.

An X-ray crystal structure of a dimeric complex (PDB ID: 1FQ9) confirmed two essential prerequisites for FGFR activation: the involvement of two molecules of FGFR that become dimers and two molecules of heparins [30]. The molecules involved in FGFR activation were heparan sulfate proteoglycans (HSPGs) on the cell membrane, and the two heparins used in the crystal were only convenient for crystallization in vitro [22, 52]. The X-ray crystal structure included the extracellular regions of FGFR1, FGF2, and heparin in a 2:2:2 ratio [30]. The heparin simultaneously bound two FGF2 ligands and two FGFR1 receptor molecules in the 3D structure. Taking this ternary crystal complex as the template, we performed MD simulations to study the energetics and dynamics of multiple peptide binding to the FGFR protein complex. A batch of 20 chains of P13 or M6 peptides were allowed to equilibrate around the 2:2 FGFR:HS complex with the FGF removed. During the equilibration where the complex was positionally restrained, the peptides were observed to quickly bind to the complex due to their strong electrostatic attractions with FGFR and HS molecules. Subsequently, in the classical MD simulations following the equilibration, the protein complex underwent different conformational changes induced by the bound P13 or M6 peptides. To try to understand the effects of these conformational changes on the biological function of FGFR, we compared the peptide simulations to the FGF-bound (*native*) and FGF-free (*apo*) simulations of the complex. Our simulations reveal that the conformations of the FGF-bound and FGF-free forms of the complex are different dramatically. The FGF-bound complex in equilibrium resembles its crystal structure where the D2 and D3 domains are stretched, the two HS molecules bind tight to both D2 and FGF, and importantly, the two D3 domains are bound together. The closer proximity of the D3 domains suggests that the complex structure is in its active state, in agreement with the FRET-based experimental result [43]. In contrast, in the *apo* simulation, the FGFR:HS complex, losing the FGF ligand, undergoes a large hinge motion by moving the lower D3 domain of an FGFR chain towards the upper D2 domain of the opposite FGFR chain. This motion occurred spontaneously and was completed within a short simulation time of 200 ns. Thereafter, the FGF binding site remains partially closed. The *native* and *apo* simulations reveal that the D2-D3 conformations and D3-D3 domain contact are distinct for the activated FGFR and its inactivated counterpart.

We compared the structural changes and energetics of the FGFR:HS complex upon binding of P13 and M6 in the peptide simulations to the *native* and *apo* simulations. The RMSD and PCA results suggest that both peptides have the capability to maintain conformation of the complex to some intermediate forms between the active and inactive states of FGFR. Importantly, both peptides entail closer domain-domain contact of D3 that might be crucial to the signal transduction function of FGFR. It is noteworthy that the structure of the M6-bound FGFR resembles closer to the activated FGFR compared to P13 presumably due to its higher affinity to the FGFR:HS complex. Hence, the computational results provide some insights on the peptide-binding modes of P13 and M6 to FGFR and support the better proliferation activity of M6 that is concluded from our biological experiments.

The TGFβ receptors are a family of Ser/Thr kinase receptors involved in the TGFβ signaling pathway, and over thirty members determine their multifunctional nature [15]. The main functions of the TGFβ receptors include self-renewal of embryonic stem cells, gastrulation, differentiation, organ morphogenesis, and adult tissue homeostasis. The TGFβ superfamily receptors send signals to a family of intracellular Smad protein kinases that are further passed into the nucleus to regulate the expression of related genes [53]. The activation mechanism of the TGFβ receptor can be divided into three parts [54] (Supplementary Fig. S5). First, TGFβ directly binds to a constitutively active kinase TGFβ receptor II; second, TGFβ is recognized by receptor I, and receptor II recruits receptor I to form a tetramer complex; and third, TGFβ receptor II phosphorylates receptor I and then transmits the signal to the downstream substrate. An in-depth study of the TGFβ receptor activation process revealed that glycoproteins anchored on the cell membrane are involved in the activation process, including the membrane-anchored Betaglycan and Endoglin, and facilitate ligand-binding to the TGFβ receptors [55–57].

The extracellular structure of the TGFβ receptor possesses a similar structure to the three-finger toxin [58], and the extracellular structure of FGFR has immunoglobulin-like scaffolds [47]. Although there are some differences between the extracellular structures of these two receptors, one of the common features is the participation of extracellular proteoglycans with separate domains when the signaling pathway is activated [20, 57, 59]. The activation pattern of FGFR is to “pull/drag” the two monomers receptors into a dimer, and the activation mode of the TGFβ receptor is to dimerize a TGFβ receptor II homodimer and a TGFβ receptor I homodimer into a heterotetramer that can activate the downstream signaling pathway. Therefore, we hypothesized that any molecule has the ability to dimerize the FGFR and TGFβ receptors and activate their corresponding signaling pathways. So we speculate that underlying molecular mechanisms of venom peptide P13 and its mutant M6 have a higher affinity to heparan sulfate and the ability to synergize proteoglycans to activate the TGF and FGF signaling pathways.

After millions of years of evolution, venom peptides and other molecules originating from venom studied till date have resulted in a diverse and versatile drug library for further development and their utilization against diseases, including exenatide (type 2 diabetes mellitus drug), captopril (hypertension drug), and hemocoagulase (for the treatment of prophylaxis and hemorrhages during surgery) [60].

## Conclusions

This study served to broaden the overall understanding of the versatility of toxin peptides and provide more ideas on optimizing the best culture media condition(s) for culturing human stem cells. To the best of our knowledge, P13 (or its potent mutant M6) reported in this study is the first venom-based peptide that can work on the FGF and TGF-β signaling pathways to promote the self-renewal capability of hESCs.

## Supplementary information

**Additional file 1: Table S1.** The primary structure and molecular weights of P13 and its mutants. **Table S2**. Primer sequences are listed. **Table S3**. Main simulation systems investigated in this study. **Figure S1.** The signaling pathways for the pluripotency of hESCs. **Figure S2.** Root-mean-squared deviation (RMSD) analysis of FGFR in simulation systems with 10, 20, or 30 chains of P13 (left) and M6 (right). **Figure S3.**. Non-bonded interaction energies (Coulombic potential for electrostatic interaction and Lennard-Jones potential for van der Waals interaction) between FGFR and peptide (PEP), HS, and PEP. **Figure S4.** Sequence alignment of FGFRs: FGFR1, FGFR2, FGFR3, and FGFR4. **Figure S5.** Sequence alignment of TGFR1 and TGFR2.

## Data Availability

All data generated or analyzed during this study are included in this published article (and its supplementary information files).

## Abbreviations

hESCs: Human embryonic stem cells
hPSCs: Human pluripotent stem cells
iPS cells: Induced pluripotent stem cells
E8: Essential 8™
ANOVA: One-way analysis of variance
qPCR: Quantitative polymerase chain reaction
CPP: Cell-penetrating peptide
FGFRs: Fibroblast growth factor receptors
HSPGs: Heparan sulfate proteoglycans
TGFβ: Transforming Growth Factor beta
