# Supplementary material for: Novel venom-based peptides (P13 and its derivative—M6) to maintain self-renewal of human embryonic stem cells by activating FGF and TGFβ signaling pathways
Source: Stem Cell Res Ther. 2020 Jun 18;11:243. doi: 10.1186/s13287-020-01766-9 (PMC7302175; doi:10.1186/s13287-020-01766-9)
Supplement: Supplementary file 1 — Additional file 1: Table S1. The primary structure and molecular weights of P13 and its mutants. Table S2. Primer sequences are listed. Table S3. Main simulation systems investigated in this study. Figure S1. The signaling pathways for the pluripotency of hESCs. Figure S2. Root-mean-squared deviation (RMSD) analysis of FGFR in simulation systems with 10, 20, or 30 chains of P13 (left) and M6 (right). Figure S3.. Non-bonded interaction energies (Coulombic potential for electrostatic interaction and Lennard-Jones potential for van der Waals interaction) between FGFR and peptide (PEP), HS, and PEP. Figure S4. Sequence alignment of FGFRs: FGFR1, FGFR2, FGFR3, and FGFR4. Figure S5. Sequence alignment of TGFR1 and TGFR2. [file 13287_2020_1766_MOESM1_ESM.docx]

***Supporting Information***

**Novel venom-based peptides (P13 & its derivative – M6) to maintain self-renewal of human embryonic stem cells by activating FGF and TGFβ signaling pathways**

**Rui MA** **^1,2*^, Zhili REN ^1,3*^, Bin LI^1,2^, Shirley W. I. SIU^4^, Guokai CHEN^1,3^, and Hang Fai KWOK^1,2#^**

^1^ Institute of Translational Medicine, Faculty of Health Sciences, University of Macau, Avenida de Universidade, Taipa, Macau SAR

^2^ Cancer Centre, Faculty of Health Sciences, University of Macau, Avenida de Universidade, Taipa, Macau SAR

^3^ Centre of Reproduction, Development & Aging, Faculty of Health Sciences, University of Macau, Avenida de Universidade, Taipa, Macau SAR

^4^ Department of Computer and Information Science, Faculty of Science and Technology University of Macau, Avenida de Universidade, Taipa, Macau SAR

* Rui MA and Zhili REN are contributed equally to this work

^#^Address correspondence to hfkwok@um.edu.mo; Room 4006, Faculty of Health Sciences, University of Macau (E12), Avenida de Universidade, Taipa, Macau SAR

**Table S1 The primary structure and molecular weights of P13 and its mutants**

| **Name of peptides** | **MW(Da)** | **Primary sequence** |
| --- | --- | --- |
| P13 | 2194.6 | W C Y K L P D R V S I K E K G R C N |
| P13-M5 (K12E/E13K) | 2194.6 | W C Y K L P D R V S I E K K G R C N |
| P13-M6 (K12E/E13R/K14R) | 2250.6 | W C Y K L P D R V S I E R R G R C N |
| P13-M10 (C2R/K12E/E13R/K14R) | 2303.7 | W R Y K L P D R V S I E R R G R C N |
| P13-M11 (C2R/D7K/K12E/E13R/K14R) | 2316.7 | W R Y K L P K R V S I E R R G R C N |

**Table S2 Primer sequences are listed.**

| **Primer name** | **Primer sequence (5' to 3')** |
| --- | --- |
| GAPDH-F | ACATCGCTCAGACACCATG |
| GAPDH-R | TGTAGTTGAGGTCAATGAAGGG |
| POU5F1-F | CTTGAATCCCGAATGGAAAGGG |
| POU5F1-R | GTGTATATCCCAGGGTGATCCTC |
| NANOG-F | TTTGTGGGCCTGAAGAAAACT |
| NANOG-R | AGGGCTGTCCTGAATAAGCAG |
| SOX2-F | AGTGTTTGCAAAAGGGGGAAAGTAG |
| SOX2-R | CCGCCGCCGATGATTGTTATTATT |

**Table S3 Main simulation systems investigated in this study.**

| **System** | **Box length (X, Y, Z)** | **No. of atoms** | **Water** | **Ions** | **Peptide** |
| --- | --- | --- | --- | --- | --- |
| FGFR:HS | 12.9, 12.9, 12.9 | 197058 | 63248 | 22/- | - |
| FGF:FGFR:HS | 12.9, 12.9, 12.9 | 201278 | 63248 | -/- | - |
| P13:FGFR:HS | 12.7, 12.7, 12.7 | 209153 | 65287 | -/58 | 20 |
| M6:FGFR:HS | 12.7, 12.7, 12.7 | 208930 | 65186 | -/58 | 20 |


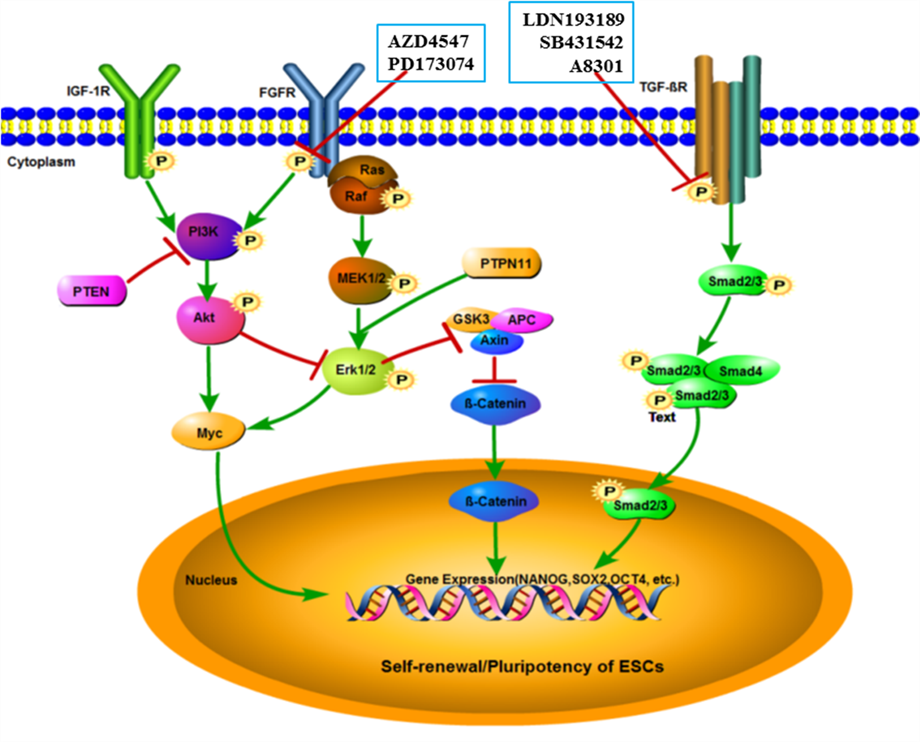


**Fig. S1 The signaling pathways for the pluripotency of hESCs.** In hESCs, TGFβ and FGFR signaling pathways are the major pathways involved in pluripotency and self-renewal; the TGFβ signaling pathway activates the Smad2/3, which can be interfered with or blocked by special small molecules TGFβ/Smad inhibitors such as LDN-193189, SB431542, and A8301. The FGFR signaling pathway activates the Akt and MAPK pathways, which can be blocked by FGFR inhibitors such as AZD4547, PD173074, and more. These signaling pathways support the pluripotent state of the hESCs, relying primarily on three key transcription factors, NANOG, SOX2, and OCT4. *Figure source adopted from ‘https://www.cellsignal.com/’ & modified by Rui MA.

**Fig. S2 Root-mean-squared deviation (RMSD) analysis of FGFR in simulation systems with 10, 20, or 30 chains of P13 (left) and M6 (right).** The protein exhibited high stability in 20-chain peptide systems for both P13 and M6; there are similar stability in 30-chain peptide system for M6.

**Fig. S3 Non-bonded interaction energies (Coulombic potential for electrostatic interaction and Lennard-Jones potential for van der Waals interaction) between FGFR and peptide (PEP), HS, and PEP.** Energies were averaged from last 100 ns of the P13 and M6 simulation trajectories.

**
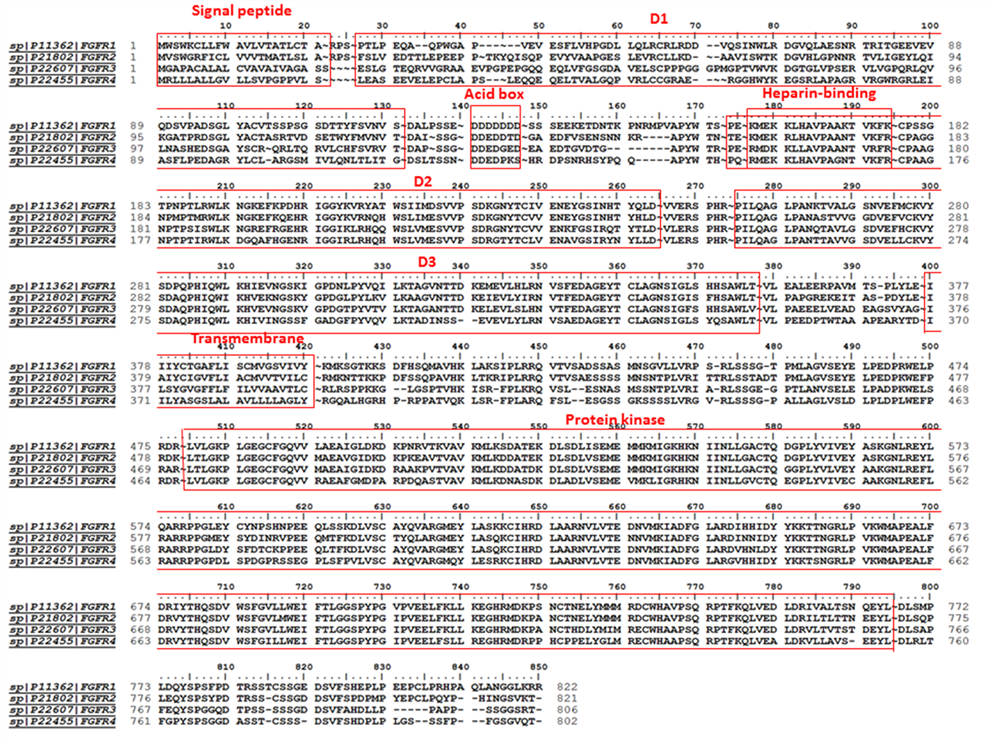
**

| **Name** | **Length**  **（AA）** | **Signal peptide** | **D1** | **D2** | **D3** | **Kinase domain** | **Heparin-binding** |
| --- | --- | --- | --- | --- | --- | --- | --- |
| FGFR1 | 822 | 1-21 | 25-119 | 158-246 | 255-357 | 468-767 | 160-177 |
| FGFR2 | 821 | 1-21 | 25-125 | 154-247 | 256-358 | 481-770 | 161-178 |
| FGFR3 | 806 | 1-22 | 23-126 | 151-244 | 253-355 | 472-761 | ——^#^ |
| FGFR4 | 802 | 1-21 | 22-118 | 152-240 | 249-349 | 467-755 | —— |

# ^#^ represents unannotated

# Fig. S4 Sequence alignment of FGFRs: FGFR1, FGFR2, FGFR3, and FGFR4. The signal peptide, acid box, heparin-binding, D1–D3 of the extracellular region, transmembrane, and kinase domain are highlighted in red. * The table is an annotation for Fig. S3, and the data are from UniProt (https://www.uniprot.org/).


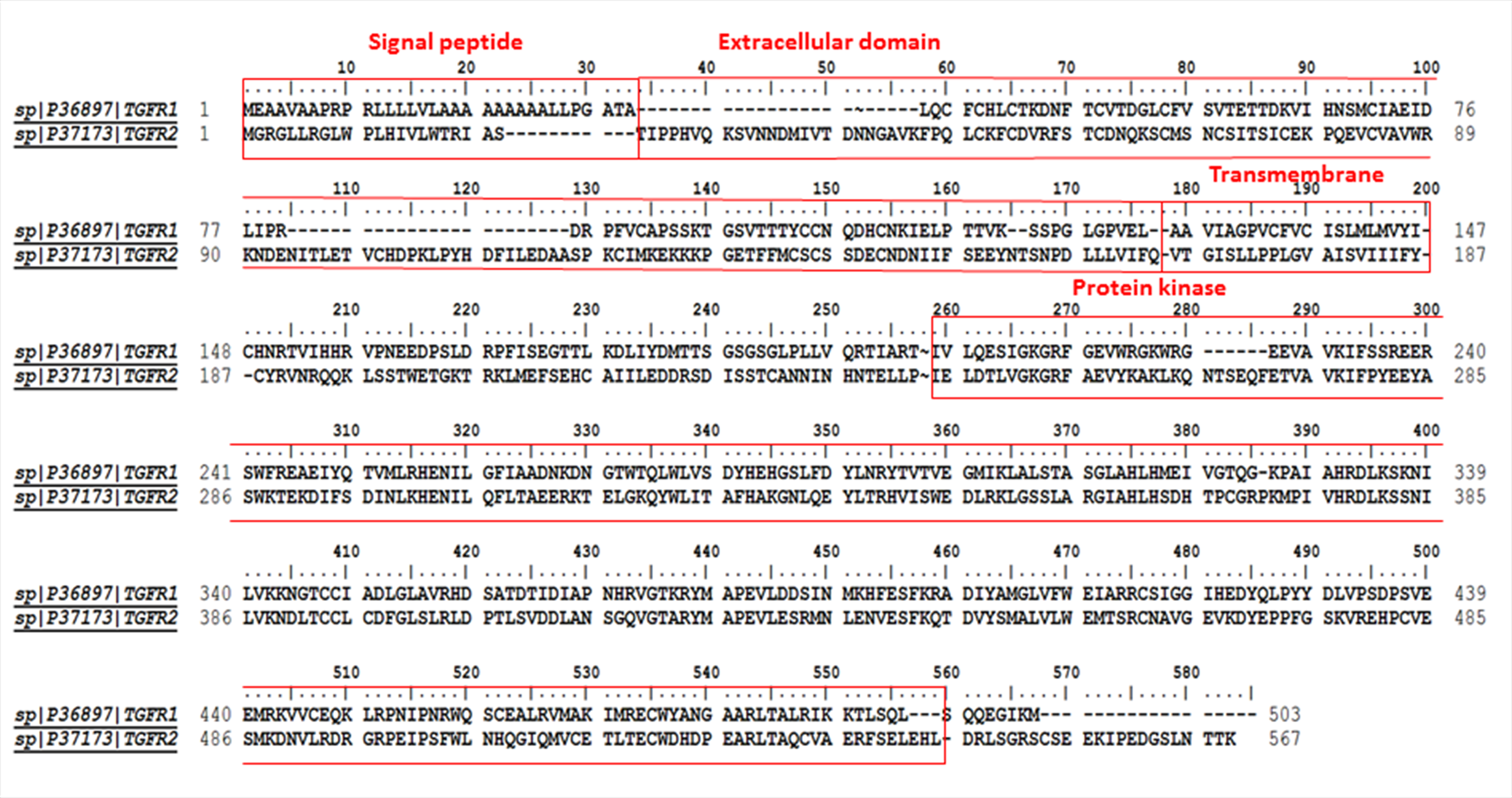


| **Name** | **Length（AA）** | **Signal peptide** | **Extracellular** | **Transmembrane domain** | **Kinase domain** |
| --- | --- | --- | --- | --- | --- |
| TGFβR1 | 503 | 1-33 | 34-126 | 127-147 | 205-495 |
| TGFβR1 | 567 | 1-22 | 23-166 | 167-187 | 244-544 |

**Fig. S5 Sequence alignment of TGFR1 and TGFR2.** The signal peptide, extracellular domain, transmembrane, and protein kinase domain are highlighted in red. * The table is an annotation for Fig. S4, and the data are from UniProt (https://www.uniprot.org/).
